# Thrombocytopenia and thrombocytosis are associated with different outcome in atrial fibrillation patients on anticoagulant therapy

**DOI:** 10.1371/journal.pone.0224709

**Published:** 2019-11-07

**Authors:** Yoav Michowitz, Robert Klempfner, Nir Shlomo, Ilan Goldenberg, Maya Koren-Michowitz

**Affiliations:** 1 Department of Cardiology, Jesselson Integrated Heart Center, Shaare Zedek Medical Center, Jerusalem, Israel; 2 Sackler School of Medicine, Tel Aviv University, Tel Aviv, Israel; 3 Department of Cardiology, Leviev Heart Institute, The Chaim Sheba Medical Center, Tel Hashomer, Israel; 4 Clinical Cardiovascular Research Center, Cardiology Division, Department of Medicine, University of Rochester Medical Center, Rochester, NY, United States of America; 5 Department of Hematology, Shamir Medical Center (Asaf Harofeh), Zerifin, Israel; Universite de Liege (B34), BELGIUM

## Abstract

**Background:**

Information regarding the significance of platelet (PLT) count on outcome of atrial fibrillation (AF) patients who are treated with anticoagulants is limited.

**Methods:**

We conducted a monocentric observational retrospective cohort study of AF patients treated with either warfarin (n = 6287) or non-vitamin K antagonist oral anticoagulants (NOACs) (n = 5240). Patient were divided into 3 subgroups; low, normal and high PLT for counts < 150 K/ μl, 150–450 K/ μl and > 450 K/ μl, respectively. A multivariate Cox-regression was used to evaluate the association between PLT subgroups and clinical outcomes.

**Results:**

During follow-up [median = 40.6 months (IQR 17.6–60)], mortality (HR 1.36, 95 CI 1.1–1.74, p = 0.01) and rate of myocardial infarction (MI) (HR 2.4, 95 CI 1.28–4.57, p = 0.007) were higher in patients with high compared to normal PLT. Transient ischemic attack or cerebrovascular accident (TIA/CVA) rate was lower in patients with low compared to normal PLT (HR 0.69, 95 CI 0.51–0.93, p = 0.02). A comparison between NOACs and warfarin demonstrated a significantly better clinical outcome for patients on NOACs in both the low (lower mortality rates) and normal PLT subgroup (lower mortality, TIA/CVA and systemic emboli rates). For patients on NOACs, low and high compared to normal PLT were associated with a higher combined outcome (HR 1.12, 95 CI 1–1.38, p = 0.047), and a higher systemic emboli rate (HR 7.07, 95 CI 1.66–30.25, p = 0.008), respectively.

**Conclusions:**

Abnormal PLT count is associated with different clinical outcome of AF patients on anticoagulants. Further studies are needed in order assess whether PLT level should influence strategies of anticoagulation.

## Introduction

Anticoagulation treatment is an important consideration in patients with atrial fibrillation (AF) and is usually given based on CHA_2_DS_2_-VASC score [1,2]. In recent years, several large-scale studies demonstrated the superiority of non-vitamin K antagonist oral anticoagulants (NOACs) over warfarin in terms of efficacy, safety or both [3–5]. Platelets (PLT) play an important role in hemostasis and their deficiency or hyperactivity may be associated with a tendency for bleeding and thrombosis, respectively [6,7]. Nevertheless, major studies on NOACs excluded patients with PLT count that was less than 90–100 K/ μl, and there is no information regarding patients with high PLT counts [3–5]. Recently, a study that was conducted in Korea demonstrated that the number of PLT played an important role on AF patients’ outcome [8]. Low PLT count was shown to be associated with a lower risk for stroke and a higher risk of bleeding. Moreover, combining PLT count with conventional risk factors improved stroke risk prediction. However, the vast majority of patients in that study were treated with warfarin, thus hampering the ability to translate these findings to contemporary treatment with NOACs. Thrombocytosis is most commonly secondary, and can be associated with increased risks of either thrombosis or bleeding, depending on the causative etiology [9,10]. There is no published data on high PLT count in AF patients and outcome. The purpose of the current study was to evaluate the significance of thrombocytopenia and thrombocytosis in AF patients treated with warfarin and more importantly with NOACs.

## Methods

### Patients

The study included a retrospective cohort of consecutive patients with non-valvular AF treated with either warfarin or NOACs in a tertiary medical center. Patients were included between June 2006 and January 2018. Baseline patient characteristics were retrospectively collected from coded diagnoses of hospitalization and outpatient clinic notes (S1 Table). Laboratory results were collected from digitalized medical charts. History of bleeding and alcohol consumption were included in the past history if the diagnosis appeared on the digitalized medical chart. Nevertheless, data on severity of bleeding and amount of alcohol consumption was not available. The study was approved by the Chaim Sheba Medical Center Institutional Review Board committee, and informed consent of individuals was waived as this study used retrospective data from regular care. The collected data were fully anonymized.

### Platelet count and subgroups

The results of complete blood counts were retrieved, and the mean of 2 consecutive PLT results was used to define the PLT subgroup, when available. All blood counts were done using the Beckman Coulter LH 750. Patients were divided into 3 subgroups: (1) Low PLT for PLT< 150 K/ μl (2) Normal PLT for 150 K/ μl ≤ PLT ≤450 K/ μl and (3) High PLT for PLT> 450 K/ μl. Patients in whom 1 blood sample showed low PLT and the second showed high PLT were considered as having laboratory result error and were excluded from the study.

### Follow-up

During follow up, 5 different endpoints were sorted from medical records and the national civil registry, including: all-cause mortality, myocardial infarction (MI), transient ischemic attack or cerebrovascular accident (TIA/CVA), systemic emboli defined as an acute vascular occlusion of an extremity or organ, and bleeding. Further details regarding severity and type of MI, TIA/CVA, systemic emboli or bleeding were not available from the digitalized coded diagnoses. In addition, 2 combined outcomes were defined, combined-1 in order to assess all adverse events (all 5 endpoints) as one outcome, and combined-2 which excluded mortality, since it may not be related to the PLT count or the anticoagulant treatment. Patients that did not have follow-up of at least 10 days were excluded from the current study.

### Statistical analysis

Continuous variables were evaluated for normal distribution using histograms and Q-Q plots. Continuous and ordinal variables are shown as mean and standard deviation (SD) or median and interquartile range [IQR] and nominal variables as n (%).

Differences in nominal variables between groups were assessed using a Pearson's chi-square test. Differences in continuous and ordinal variables between groups were assessed using independent samples T test, analysis of variance, Mann-Whitney U test or Kruskal-Wallis test.

Length of follow-up was evaluated using reverse censoring method. Kaplan-Meier curve was used to describe events during the follow-up and log rank test was used to compare between nominal variables. Multivariate Cox regression was used to evaluate the association between the variables groups and outcomes after controlling for potential confounders. Age and gender were forced into the regression while history of CHF, DM, HTN, PVD, DVT/PE, IHD, fall, alcohol consumption, bleeding, use of aspirin, use of clopidogrel, Hemoglobin (Hb), white blood cell count (WBC), mean platelet volume (MPV) and glomerular filtration rate (GFR) were initially included and then were considered for removal using backward section methods (Wald test was used and p> 0.1 was set as criteria for removal). In the multivariate analysis 5.2% of patients were dropped out due to missing data. Since this rate is low we preferred to use raw data rather than using imputation methods.

Significant p values were considered when p<0.05. All calculations were done using SPSS version 25 (IBM, Armonk, NY, USA).

## Results

After exclusion of 359 patients with either no follow-up (n = 109), no blood count sample (n = 239) or error in blood test results (i.e. 1 blood sample showing low PLT and the second showing high PLT) (n = 11), the study included 11527 patients, treated with either warfarin (n = 6287) or NOACs (n = 5240). NOACs included dabigatran (n = 951), rivaroxaban (n = 1330) and apixaban (n = 2959). Out of 11527 patients, 493 (4.3%) had one PLT measurement, while 95.7% of patients had 2 measurements. Patients were divided into 3 subgroups as defined above including: patients with low PLT (n = 1617, median PLT 129.5, IQR 114.5–141 K/ μl, 189 (11.7%) of them with PLT≤100,000), patients with normal PLT (n = 9739, median PLT 219.5, IQR 186.5–266.5 K/ μl) and patients with high PLT (n = 171, median PLT 508, IQR 470.5–577 K/ μl). Table 1 describes baseline patients characteristics. The rates of missing data are shown in S2 Table. Patients with low PLT were older, with a higher proportion of males and past history of IHD or CHF. Past history of DVT/PE was seen more often in patients with high PLT. Ejection fraction (EF) was higher in the normal PLT group, creatinine level was higher in patients with low PLT, and patients with high PLT had abnormal additional blood count measures (lower Hb, higher WBC and lower MPV). Differences in other baseline laboratory results are further described in the Table.

**Table 1:** Baseline characteristics of PLT subgroups.

	Low platelets	Normal platelets	High platelets	P value
	n = 1617	n = 9739	n = 171	
**Age, years**	78.7 (71, 84.5)	76.6 (67.9, 83.4)	76.6 (65.7, 83.4)	**<0.001**^b^
**Male**	1163 (71.9)	4893 (50.2)	83 (48.5)	**<0.001**^**c**^
**Past history**				
**CHF**	579 (35.8)	2846 (29.2)	45 (26.3)	**<0.001**^**c**^
**DM**	475 (29.4)	2862 (29.4)	64 (37.4)	0.07^c^
**Hypertension**	1036 (64.1)	6166 (63.3)	110 (64.3)	0.81^c^
**PVD**	95 (5.9)	492 (5.1)	12 (7)	0.22^c^
**Past PE/DVT**	64 (4)	456 (4.7)	17 (9.9)	**<0.001**^**c**^
**IHD**	766 (47.4)	3817 (39.2)	71 (41.5)	**<0.001** ^c^
**Fall**	146 (9)	937 (9.6)	17 (9.9)	0.74 ^c^
**Alcohol consumption**	43 (2.7)	212 (2.2)	2 (1.2)	0.3 ^c^
**TIA/CVA**	338 (20.9)	2252 (23.1)	39 (22.8)	0.14 ^c^
**Bleeding**	123 (7.6)	653 (6.7)	8 (4.7)	0.22 ^c^
**Aspirin**	549 (34)	3457 (35.5)	58 (33.9)	0.45 ^c^
**Clopidogrel**	148 (9.2)	999 (10.3)	17 (9.9)	0.39 ^c^
**Weight, Kg**	75 (67, 87.3)	75.3 (65, 87.7)	72 (61, 86)	**0.032** ^b^
**CHADS2 score**	2 (1, 3)	2 (1, 3)	2 (1, 3)	**0.01** ^b^
**CHA2DS2-VASC score**	4 (3, 5)	4 (3, 5)	4 (3, 6)	0.89 ^b^
**Laboratory results**				
**EF, %**	55 (40, 60)	60 (50, 60)	55 (45, 60)	**<0.001** ^b^
**Creatinine, mg/dL**	1.1 (0.9, 1.4)	1 (0.8, 1.3)	1 (0.8, 1.3)	**<0.001** ^b^
**Hb, g/dL**	12.2±1.8	12.2±1.8	11.1±1.7	**<0.001**^**a**^
**WBC, 10**^**9**^**/L**	7.1 (5.7, 9)	8.8 (7, 11)	11.9 (9.4, 15)	**<0.001** ^b^
**MPV, fL**	9.6 (8.8, 10.6)	8.8 (8.1, 9.6)	7.8 (7, 8.4)	**<0.001** ^b^
**GFR, mL/min**	59.2 (44.6, 73.6)	62 (48, 78)	63.5 (50.9, 85.6)	**<0.001** ^b^

CHF = congestive heart failure; DM = diabetes mellitus; PVD = peripheral vascular disease; PE/DVT = pulmonary emboli/ deep vein thrombosis; IHD = ischemic heart disease; TIA/CVA = transient ischemic attack/ cerebrovascular accident; EF = ejection fraction; Hb = hemoglobin, WBC = white blood cells; MPV = mean platelet volume; GFR = glomerular filtration rate.

^a^ANOVA

^b^Kruskal-Wallis test

^c^Chi square test

### Outcomes according to platelet count

**Fig 1:**
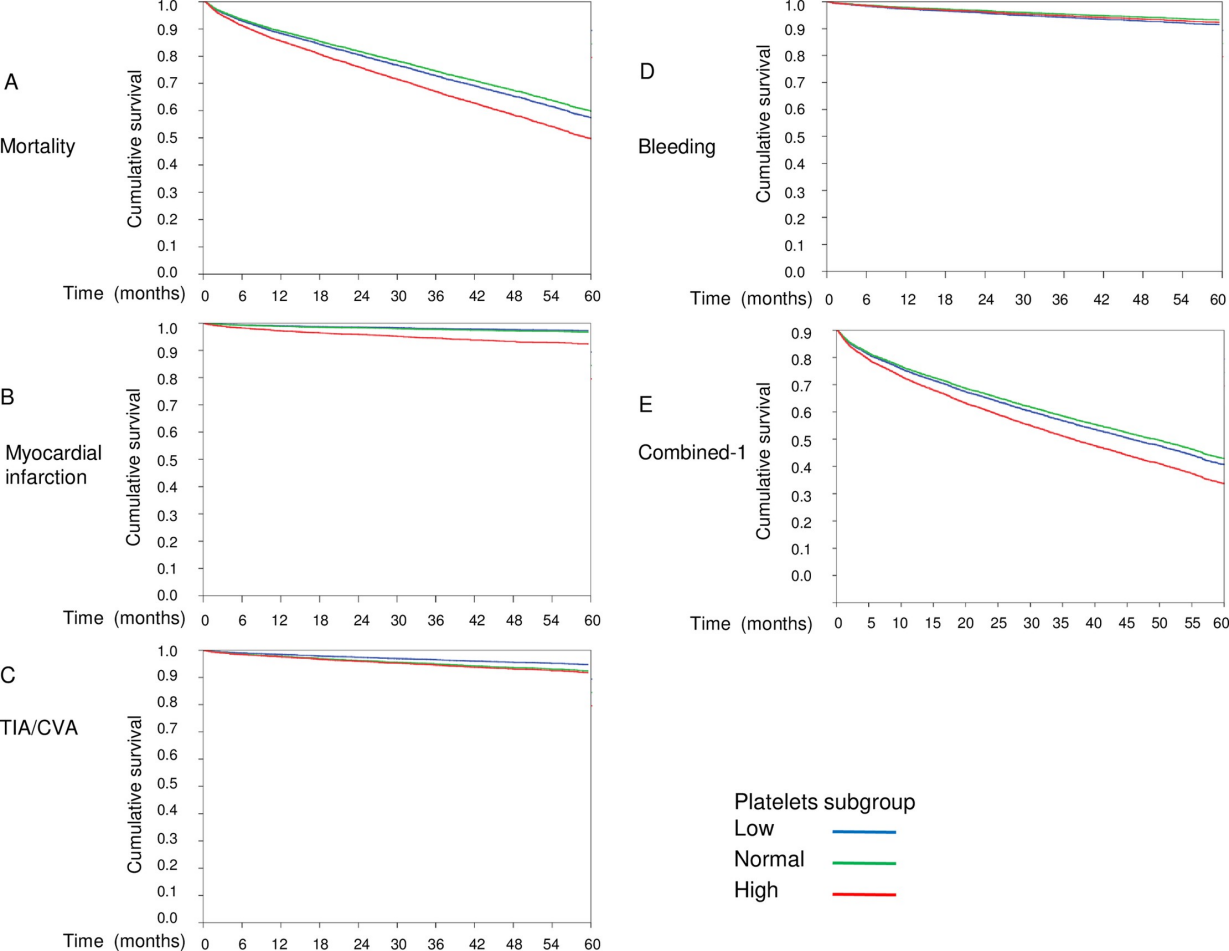
Kaplan-Meier curves for clinical endpoints in patients with low, normal and high platelet (PLT) count. (A) Overall survival. Patients with high PLT (red line) had significantly higher mortality rate during follow up. (B) Myocardial infarction free survival. Patients with high PLT had significantly higher rates of myocardial infarction during follow up. (C) Transient ischemic attack/ cerebrovascular accident (TIA/CVA) free survival. Patients with low PLT (blue line) compared to patients with normal PLT had significantly lower rates of TIA/CVA. (D) Bleeding free survival. No significant difference in bleeding episodes was seen between the 3 groups. A tendency for higher bleeding rate was seen in patients with low compared to normal PLT. (E) Combined-1 free survival. The rate of the combined-1 outcome that included mortality, MI, TIA/CVA, systemic emboli and bleeding was significantly higher among patients with high PLT.

Median follow-up time was 40.6 months (IQR 17.6–60 months). The cumulative survival at 1, 12, 24, 36, 48 and 60 months was 98.4%, 85.9%, 77.6%, 70%, 63% and 56.2%, respectively and the number of patients at risk at these time points was 11219, 8199, 6011, 4397, 3126 and 2248, respectively. The number of patients with each outcome type by PLT and treatment groups is given in S3 Table. As baseline characteristics were different between the various subgroups a multivariate cox regression was used to evaluate the significance of outcomes after controlling for potential confounders. Since substantial number of patients (37%) did not have data on EF, this variable was not included in the multivariate analysis. Data for all other studied variables was available in 93.5–100% of the patients. Table 2 summarizes the results of the multivariate cox regression. Mortality (HR 1.36, 95 CI 1.1–1.74, p = 0.01) and the rate of MI (HR 2.4, 95 CI 1.28–4.57, p = 0.007) were higher in patients with high compared to normal PLT. TIA/CVA rate during follow up was lower in patients with low compared to normal PLT (HR 0.69, 95 CI 0.51–0.93, p = 0.02). Bleeding rate tended to be higher in patients with low compared to normal PLT (HR-1.27, 0.99–1.62, p = 0.054). Both combined endpoints were higher in patients with high compared to normal PLT. Kaplan-Meier curves comparing the above-mentioned differences as well as for the combined-1 outcome are presented in Fig 1.

**Table 2:** Multivariate cox regression analysis for clinical outcomes.

Outcome*	Low vs. normal platelets			High vs. normal platelets			All Groups
	HR	95 CI	P value	HR	95 CI	P value	P value**
**Mortality**^**a**^	1.08	0.98–1.19	0.1	1.36	1.1–1.74	**0.01**	**0.02**
**MI**^**b**^	0.84	0.6–1.17	0.3	2.4	1.28–4.57	**0.007**	**0.01**
**TIA/CVA**^**c**^	0.69	0.51–0.93	**0.02**	1.08	0.54–2.18	0.8	0.051
**Systemic Emboli**^**d**^	1.16	0.69–1.93	0.57	1.43	0.45–4.53	0.54	0.72
**Bleeding**^**e**^	1.27	0.99–1.62	0.054	1.14	0.56–2.29	0.72	0.15
**Combined-1**^**f**^	1.07	0.98–1.17	0.15	1.3	1.03–1.64	**0.03**	**0.04**
**Combined-2**^**g**^	0.99	0.84–1.17	0.92	1.53	1.02–2.27	**0.04**	0.11

MI = myocardial infarction; TIA/CVA = transient ischemic attack/ cerebrovascular accident; Combined-1 includes: mortality, MI, TIA/CVA, systemic emboli and bleeding; Combined-2 includes: MI, TIA/CVA, systemic emboli and bleeding.

*Parameters included in the final multivariate model for

^a^ Age, Gender, CHF, DM, Hypertension

^b^Age, Gender, DM, IHD, Clopidogrel, GFR, Hb, WBC

^c^Age, Gender, Alcohol consumption, past TIA/CVA, GFR

^d^Age, Gender, past PE/DVT, Aspirin, WBC

^e^Age, Gender, CHF, DM, Hypertension, past PE/DVT, Bleeding, GFR, Hb, WBC

^f^Age, Gender, CHF, DM, Hypertension, PVD, past PE/DVT, IHD, Fall, past TIA/CVA, Bleeding, Aspirin, GFR, Hb, WBC

^g^Age, Gender, CHF, DM, Hypertension, past PE/DVT, IHD, Alcohol consumption, past TIA/CVA, Bleeding, Clopidogrel, GFR, WBC

**p value for comparison of the 3 patients’ subgroups.

### Comparison of NOACs and Warfarin in the various platelet count subgroups

Table 3 presents baseline characteristics of patients treated with NOACs compared to warfarin in the various PLT subgroups. In patients with low PLT, those treated with NOACs were older, and a higher percentage of patients had past history of DM, HTN, IHD, TIA/CVA, previous fall and bleeding. More patients on NOACs were also treated with clopidogrel, and had higher CHADS_2_ or CHA_2_DS_2_-VASC scores. In the normal PLT subgroup, compared to warfarin, patient on NOACs were older, a higher percentage of patients had past history of CHF, DM, HTN, PVD, IHD, TIA/CVA, previous fall and bleeding. More patients on NOACs were also treated with clopidogrel, and had higher CHADS_2_ or CHA_2_DS_2_-VASC scores. In the high PLT subgroup, patients treated with NOACs compared to warfarin were older, had more often history of DM and HTN and had higher CHADS_2_ or CHA_2_DS_2_-VASC scores.

**Table 3:** Baseline characteristics of patients treated with warfarin vs. non-vitamin K antagonist oral anticoagulants (NOACs).

	Low platelet			Normal platelet			High platelet		
	Warfarin	NOAC	p value	Warfarin	NOAC	p value	Warfarin	NOAC	p value
	n = 905	n = 712		n = 5272	n = 4467		n = 110	n = 61	
**Age, years**	77.7 (70.1, 83.7)	79.7 (71.9, 85.5)	**<0.001**^c^	75.6 (66.4, 82.7)	77.7 (69.5, 84.4)	**<0.001** ^c^	72.2 (64, 81.1)	79.5 (70.6, 85.5)	**0.001** ^c^
**Male**	654 (72.3)	509 (71.5)	0.74^a^	2672 (50.7)	2221 (49.7)	0.35 ^a^	56 (50.9)	27 (44.3)	0.43 ^a^
**Medical history**									
**CHF**	320 (35.4)	259 (36.4)	0.67 ^a^	1471 (27.9)	1375 (30.8)	**0.02** ^a^	24 (21.8)	21 (34.4)	0.1 ^a^
**DM**	233 (25.7)	242 (34)	**<0.001** ^a^	1375 (26.1)	1486 (33.3)	**<0.001** ^a^	35 (31.8)	29 (47.5)	**0.049** ^a^
**Hypertension**	527 (58.2)	509 (71.5)	**<0.001** ^a^	3054 (57.9)	3112 (69.7)	**<0.001** ^a^	57 (51.8)	53 (86.9)	**<0.001** ^a^
**PVD**	46 (5.1)	49 (6.9)	0.14 ^a^	234 (4.4)	258 (5.8)	**0.003** ^a^	7 (6.4)	5 (8.2)	0.76 ^a^
**Past DVT/PE**	37 (4.1)	27 (3.8)	0.8 ^a^	254 (4.8)	202 (4.5)	0.5 ^a^	12 (10.9)	5 (8.2)	0.6 ^a^
**IHD**	403 (44.5)	363 (51)	**0.01** ^a^	1945 (36.9)	1872 (41.9)	**<0.001** ^a^	44 (40)	27 (44.3)	0.62 ^a^
**Fall**	53 (5.9)	93 (13.1)	**<0.001** ^a^	303 (5.7)	634 (14.2)	**<0.001** ^a^	9 (8.2)	8 (13.1)	0.42 ^a^
**Alcohol**	19 (2.1)	24 (3.4)	0.12 ^a^	106 (2)	106 (2.4)	0.24 ^a^	2 (1.8)	0 (0)	0.53 ^a^
**TIA/CVA**	155 (17.1)	183 (25.7)	**<0.001** ^a^	993 (18.8)	1259 (28.2)	**<0.001** ^a^	22 (20)	17 (27.9)	0.26 ^a^
**Bleeding**	51 (5.6)	72 (10.1)	**0.001** ^a^	253 (4.8)	400 (9)	**<0.001** ^a^	4 (3.6)	4 (6.6)	0.46 ^a^
**Aspirin**	304 (33.6)	245 (34.4)	0.75 ^a^	1914 (36.3)	1543 (34.5)	0.07 ^a^	41 (37.3)	17 (27.9)	0.24 ^a^
**Clopidogrel**	56 (6.2)	92 (12.9)	**<0.001** ^a^	411 (7.8)	588 (13.2)	**<0.001** ^a^	7 (6.4)	10 (16.4)	0.06 ^a^
**Weight, Kg**	75 (66.9, 87)	76 (68, 89)	0.09 ^c^	75 (65, 87)	76 (66, 88)	**0.001** ^c^	73.7 (62.5, 86)	71 (60.5, 87.7)	0.5 ^c^
**CHADS2 score**	2 (1, 3)	3 (2, 4)	**<0.001** ^c^	2 (1, 3)	2 (1, 4)	**<0.001** ^c^	2 (1, 3)	3 (2, 4)	**<0.001** ^c^
**CHA2DS2-VASC score**	4 (2, 5)	4 (3, 5)	**<0.001** ^c^	4 (2, 5)	4 (3, 6)	**<0.001** ^c^	3 (2, 5)	5 (3, 6)	**<0.001** ^c^
**Laboratory**									
**EF, %**	55 (35, 60)	55 (42, 60)	**<0.001** ^c^	55 (45, 60)	60 (50, 60)	**<0.001** ^c^	56.5 (46.3, 60)	50 (45, 60)	0.57 ^c^
**Creatinine, mg/dL**	1.2 (1, 1.5)	1 (0.9, 1.3)	**<0.001** ^c^	1.1 (0.9, 1.4)	1 (0.8, 1.2)	**<0.001** ^c^	1.1 (0.8, 1.3)	0.8 (0.7, 1.2)	**<0.001**^c^
**Hb, g/dL**	12.1±1.9	12.4±1.8	**0.001**^b^	12.1±1.9	12.4±1.8	**<0.001** ^b^	11.3±1.7	10.9±1.5	0.1 ^b^
**WBC, 10**^**9**^**/L**	7.1 (5.7, 9.2)	7.2 (5.8, 9)	0.6 ^c^	8.8 (7.1, 11)	8.8 (7.1, 11)	0.86 ^c^	12.2 (9.7, 15.6)	11.1 (8.3, 14.4)	0.07 ^c^
**MPV, fL**	9.6 (8.7, 10.5)	9.7 (8.9, 10.7)	**0.03** ^c^	8.8 (8.1, 9.6)	8.8 (8.1, 9.7)	**0.02** ^c^	7.8 (7, 8.3)	7.8 (7.1, 8.5)	0.88 ^c^
**GFR, mL/min**	55.6 (41.4, 68.3)	64 (49.3, 81.6)	**<0.001** ^c^	58.2 (44.7, 71.9)	67.8 (52.5, 84.9)	**<0.001** ^c^	59.8 (49.4, 70.6)	74.3 (53.8, 104.5)	**0.002** ^c^

CHF = congestive heart failure; DM = diabetes mellitus; PVD = peripheral vascular disease; PE/DVT = pulmonary emboli/ deep vein thrombosis; IHD = ischemic heart disease; TIA/CVA = transient ischemic attack/ cerebrovascular accident; EF = ejection fraction; Hb = hemoglobin, WBC = white blood cells; MPV = mean platelet volume; GFR = glomerular filtration rate.

^a^chi square test

^b^Independent sample T test

^c^Mann-Whitney test

### Outcomes according to treatment with NOACs or warfarin in the various platelet count subgroups

A multivariate cox regression analysis demonstrated that in the low PLT subgroup, mortality was lower in patients treated with NOACs compared to warfarin (HR 0.78, 95 CI 0.64–0.96, p = 0.02) (Table 4). In the normal PLT subgroup treatment with NOACs was associated with lower mortality (HR 0.69, 95 CI 0.63–0.76, p<0.001), TIA/CVA (HR 0.8, 95 CI 0.65–0.99, p = 0.04), systemic emboli (HR 0.6, 95 CI 0.38–0.96, p = 0.03) and in the combined-1 outcome (HR 0.76, 95 CI 0.69–0.82, p<0.001). In the high PLT subgroup, low number of events precluded subgroup analysis for MI, TIA/CVA, systemic emboli, bleeding and combined-2. There was no difference in outcome in mortality and combined-1 outcomes.

**Table 4:** Multivariate cox regression analysis for clinical outcomes in patients treated with non-vitamin K antagonist oral anticoagulants (NOACs) compared to warfarin.

	Low platelet			Normal platelet			High platelet		
Outcome*	HR	95 CI	P value	HR	95 CI	P value	HR	95 CI	P value
**Mortality**^a^	0.78	0.64–0.96	**0.02**	0.69	0.63–0.76	**<0.001**	0.64	0.35–1.2	0.14
**MI**^b^	1.8	0.95–3.4	0.07	0.93	0.7–1.2	0.59	**		
**TIA/CVA**^c^	1.2	0.68–2,2	0.5	0.8	0.65–0.99	**0.04**	**		
**Systemic Emboli**^d^	**			0.6	0.38–0.96	**0.03**	**		
**Bleeding**^e^	0.95	0.58–1.56	0.83	0.89	0.7–1.12	0.33	**		
**Combined-1**^f^	0.94	0.78–1.13	0.54	0.76	0.69–0.82	**<0.001**	0.71	0.41–1.23	0.22
**Combined-2**^g^	1.3	0.93–1.8	0.13	0.9	0.78–1.03	0.13	**		

MI = myocardial infarction; TIA/CVA = transient ischemic attack/ cerebrovascular accident; Combined-1 includes: mortality, MI, TIA/CVA, systemic emboli and bleeding; Combined-2 includes: MI, TIA/CVA, systemic emboli and bleeding.

*Parameters included in the final multivariate model: Low platelet-

^a^Age, Gender, CHF, DM, Fall, past TIA/CVA, GFR, Hb, WBC

^b^Age, Gender, Hypertension, IHD, Bleeding, GFR

^c^Age, Gender, past TIA/CVA, Aspirin

^e^Age, Gender, GFR, Hb

^f^Age, Gender, CHF, DM, Hypertension, past TIA/CVA, Bleeding, GFR, Hb, WBC

^g^Age, Gender, Hypertension, IHD, past TIA/CVA, Bleeding, Clopidogrel, GFR; Normal platelet- ^a^ Age, Gender, CHF, DM, Hypertension, PVD, past PE/DVT, IHD, Fall, past TIA/CVA, Bleeding, Aspirin, GFR, Hb, WBC; ^b^ Age, Gender, DM, PVD, IHD, Clopidogrel, GFR, Hb, WBC; ^c^ Age, Gender, Alcohol consumption, past TIA/CVA, MPV

^d^ Age, Gender, past PE/DVT, Alcohol consumption, past TIA/CVA, Aspirin, WBC; ^e^ Age, Gender, CHF, DM, PVD, past PE/DVT, Alcohol consumption, Bleeding, GFR, Hb; ^f^ Age, Gender, CHF, DM, Hypertension, PVD, past PE/DVT, IHD, Fall, past TIA/CVA, Bleeding, Aspirin, GFR, Hb, WBC; ^g^ Age, Gender, CHF, DM, Hypertension, PVD, past PE/DVT, IHD, Alcohol consumption, past TIA/CVA, Bleeding, GFR, WBC; High platelet- ^a^ Age, Gender, past PE/DVT, IHD, Clopidogrel; ^f^ Age, Gender, past PE/DVT, Clopidogrel.

**Low number of events precluded subgroup analysis

### Comparison of patients in PLT subgroups on NOACs

In order to further analyze the significance of platelet count on patients treated with contemporary anticoagulant treatment, patients on NOACs only were analyzed. Patients with low compared to normal PLT (S4 Table) were older, a higher proportion were males and more had a history of IHD and lower EF. A multivariate cox regression analysis demonstrated a significant higher combined-1 outcome in patients on NOACs with low compared to normal PLT (HR 1.12, 95 CI 1–1.38, p = 0.047). None of the different outcomes alone were significantly different between the groups (S5 Table).

Patients with high compared to normal PLT (S6 Table) were more often diabetic and hypertensive with higher CHADS_2_ and CHA_2_DS_2_-VASC scores. Differences in laboratory results are further shown in the table. A multivariate cox regression analysis demonstrated a significant higher systemic emboli outcome in patients on NOACs with high compared to normal PLT (HR 7.07, 95 CI 1.66–30.25, p = 0.008) (S7 Table).

## Discussion

The current study includes a very large cohort of AF patients on anticoagulants, 45.5% treated with NOACs and 54.5% with warfarin. It illustrates 4 main findings: 1) even mild thrombocytopenia seems protective of stroke; 2) High PLT is associated with higher rates of mortality and MI; 3) In patients with low and normal PLT, NOACs have outcome benefit compared to warfarin, and (4) In patient on NOACs, low PLT compared to normal PLT is associated with higher rates of the combined-1 outcome and high compared to normal PLT is associated with higher rates of systemic emboli.

### Low PLT and lower stroke rates

In accordance with the current study, Park and colleagues [8] demonstrated that moderate to severe thrombocytopenia below 100 K/ μl is associated with lower stroke but higher bleeding rates in AF patients. However, their study was confined almost exclusively to patients on warfarin. The current study in which almost half of its population was treated with NOACs expands these findings. It demonstrates that even mild thrombocytopenia (only 11.7% had PLT≤100 K/ μl) is associated with lower stroke rates and a tendency to higher bleeding rates. It should be emphasized that an analysis that included patients treated with NOACs only did not show significant differences of stroke and bleeding rates between patients with low and normal PLT. Whether it may be related to a smaller group of patients need to be defined. Nevertheless, the sum of all endpoints, i.e. the combined-1 endpoint was significantly higher among the NOAC low PLT group, suggesting that as NOACs may be more potent than warfarin, the protective role of low PLT against stroke is clinically insignificant in the era of NOACs. Also, low PLT may be a marker of a comorbid condition, or by itself cause of more morbid combined outcome. Of note, a previous study [11] suggested that NOACs are safe in patients with mild thrombocytopenia, however it was limited by a very small population size.

### Comparison of Warfarin and NOACs in the different PLT groups

Treatment with NOACs was shown to be superior to warfarin in several mega trials [3–5], however relationship between patients’ outcomes and PLT levels was not reported. Wang and colleagues [12] compared the safety and efficacy of NOACs compared to warfarin in patients with PLT levels higher or lower than 100 K/ μl. In patients with PLT > 100 K/ μl they demonstrated better safety (less bleeding), however in lower PLT levels they could not show statistically different outcomes as the study was limited by low patient numbers. The current study which includes a larger cohort, demonstrated that NOACs treatment is associated with lower mortality in patients with mild thrombocytopenia as well as normal PLT levels. Superiority of NOACs over warfarin could not be demonstrated in patients with high PLT levels, however their number was small (see below).

### High PLT and higher rates of MI and mortality

Thrombocytosis is most commonly reactive, with bleeding, iron deficiency, infection / inflammation and cancer being the most common associated primary diagnoses [6,7,9]. The Khorana score demonstrated that PLT > 350 K/ μl is a risk factor for venous thromboembolism in cancer patients [13,14], and thrombocytosis is also associated with arterial thrombosis in patients with the myeloproliferative neoplasms [15]. Whether it is also associated with higher risk for arterial thrombosis in other etiologies is unknown. Since we included patients with high PLT in 2 blood counts, this subgroup could be potentially enriched for patients with cancer, including myeloproliferative neoplasms. Our novel finding showing higher rates of mortality, MI and combined outcomes in patients with high compared to normal PLT is intriguing, and could be related to an underlying diagnosis. Moreover, in patients on NOACS only high PLT was associated with higher rates of systemic emboli. Yet, due to relatively low patient numbers in each subgroup category, validation is needed in larger cohorts. Nevertheless, these patients should be more closely monitored during follow up. Further studies on larger groups of patients with high PLT on warfarin and NOACS should be conducted, as well as the potential benefit of adding antiaggregant therapy to the anticoagulation.

### Limitations

The study is retrospective and baseline group characteristics were not equal, despite statistical adjustment we cannot exclude the possibility of bias of unmeasurable confounders. Outcome data was derived from coded diagnoses and diagnoses were not revised [16], which can cause bias. Bleeding appearing in the coded diagnoses were not categorized according to the BARC (Bleeding Academic Research Consortium) criteria, although we assumed that the coded diagnoses included mainly bleeding that were clinically significant (BARC 3–5) [17]. In addition, the diagnoses were collected using a digitalized coded system, thus further assessment of severity of patient baseline comorbidities and outcome was not possible. Nevertheless, all-cause mortality, a hard endpoint, was significantly different in several important comparisons. Data on patients’ compliance to anticoagulation treatment and various dose adjustments according to patients’ clinical characteristics was not available, however this is a real-world data. Additionally we could not use EF in the multivariate analysis due to a high missing rate, although we could use CHF in the model. Finally, the group of patients with thrombocytosis is heterogeneous and small and the etiology of thrombocytosis which could affect patients’ outcome was not sorted. However, this data is thought provocative and is presented not for achieving definitive conclusions but rather to suggest that more research is needed in larger and more homogeneous groups.

## Conclusion

PLT count has an important influence on outcome of AF patients treated with anticoagulants. Mild thrombocytopenia is associated with lower stroke rates and a tendency for more bleeding, and thrombocytosis is associated with more MI and higher mortality rates. In patients on NOACs only thrombocytopenia and thrombocytosis are associated with a higher combined endpoint and higher systemic emboli rates, respectively. Further research is needed in order to assess the best anticoagulation strategy for patient with AF and abnormal PLT level.

## Supporting information

S1 Table List of diagnostic codes. (DOCX)

S2 Table Rate of missing baseline data. (DOCX)

S3 Table Outcome type distribution according to PLT subgroup and anticoagulant treatment. (DOCX)

S4 Table Baseline characteristics of low and normal PLT subgroups treated with non-vitamin K antagonist oral anticoagulants (NOACs). (DOCX)

S5 Table Multivariate cox regression analysis for clinical outcomes of low compared to normal PLT subgroups treated with non-vitamin K antagonist oral anticoagulants (NOACs). (DOCX)

S6 Table Baseline characteristics of high and normal PLT subgroups treated with non-vitamin K antagonist oral anticoagulants (NOACs). (DOCX)

S7 Table Multivariate cox regression analysis for clinical outcomes of high compared to normal PLT subgroups treated with non-vitamin K antagonist oral anticoagulants (NOACs). (DOCX)
