# Supplementary material for: Thrombocytopenia and thrombocytosis are associated with different outcome in atrial fibrillation patients on anticoagulant therapy
Source: PLoS One. 2019 Nov 7;14(11):e0224709. doi: 10.1371/journal.pone.0224709 (PMC6837521; doi:10.1371/journal.pone.0224709)
Supplement: S1 Table — (DOCX) [file pone.0224709.s001.docx]

| Code | **Myocardial infarction** |
| --- | --- |
| 4100 | MYOCARDIAL INFARCTION OF ANTEROLATERAL WALL |
| 4101 | MYOCARDIAL INFARCTION - ANTERIOR WALL |
| 4102 | MYOCARDIAL INFARCTION OF INFEROLATERAL WALL |
| 4103 | MYOCARDIAL INFARCTION OF INFEROPOSTERIOR WALL |
| 4104 | MYOCARDIAL INFARCTION OF INFERIOR WALL |
| 4105 | MYOCARDIAL INFARCTION - LATERAL WALL |
| 410 | MYOCARDIAL INFARCTION |
| 410.00 | ACUTE MYOCARDIAL INFARCTION, OF ANTEROLATERAL WALL, EPISODE OF CARE UNSPECIFIED |
| 410.01 | ACUTE MYOCARDIAL INFARCTION, OF ANTEROLATERAL WALL, INITIAL EPISODE OF CARE |
| 410.02 | ACUTE MYOCARDIAL INFARCTION, OF ANTEROLATERAL WALL, SUBSEQUENT EPISODE OF CARE |
| 410.10 | ACUTE MYOCARDIAL INFARCTION, OF OTHER ANTERIOR WALL, EPISODE OF CARE UNSPECIFIED |
| 410.11 | ACUTE MYOCARDIAL INFARCTION, OF OTHER ANTERIOR WALL, INITIAL EPISODEOF CARE |
| 410.12 | ACUTE MYOCARDIAL INFARCTION, OF OTHER ANTERIOR WALL, SUBSEQUENT EPISODE OF CARE |
| 410.20 | ACUTE MYOCARDIAL INFARCTION, OF INFEROLATERAL WALL, EPISODE OF CARE UNSPECIFIED |
| 410.21 | ACUTE MYOCARDIAL INFARCTION, OF INFEROLATERAL WALL, INITIAL EPISODE OF CARE |
| 410.22 | ACUTE MYOCARDIAL INFARCTION, OF INFEROLATERAL WALL, SUBSEQUENT EPISODE OF CARE |
| 410.30 | ACUTE MYOCARDIAL INFARCTION, OF INFEROPOSTERIOR WALL, EPISODE OF CARE UNSPECIFIED |
| 410.31 | AMI INFEROPOST, INITIAL |
| 410.32 | ACUTE MYOCARDIAL INFARCTION, OF INFEROPOSTERIOR WALL, SUBSEQUENT EPISODE OF CARE |
| 410.40 | ACUTE MYOCARDIAL INFARCTION, OF OTHER INFERIOR WALL, EPISODE OF CAREUNSPECIFIED |
| 410.41 | ACUTE MYOCARDIAL INFARCTION, OF OTHER INFERIOR WALL, INITIAL EPISODEOF CARE |
| 410.42 | ACUTE MYOCARDIAL INFARCTION, OF OTHER INFERIOR WALL, SUBSEQUENT EPISODE OF CARE |
| 410.50 | ACUTE MYOCARDIAL INFARCTION, OF OTHER LATERAL WALL, EPISODE OF CARE UNSPECIFIED |
| 410.51 | ACUTE MYOCARDIAL INFARCTION, OF OTHER LATERAL WALL, INITIAL EPISODE OF CARE |
| 410.52 | ACUTE MYOCARDIAL INFARCTION, OF OTHER LATERAL WALL, SUBSEQUENT EPISODE OF CARE |
| 410.60 | ACUTE MYOCARDIAL INFARCTION, TRUE POSTERIOR WALL INFARCTION, EPISODEOF CARE UNSPECIFIED |
| 410.61 | ACUTE MYOCARDIAL INFARCTION, TRUE POSTERIOR WALL INFARCTION, INITIALEPISODE OF CARE |
| 410.62 | ACUTE MYOCARDIAL INFARCTION, TRUE POSTERIOR WALL INFARCTION, SUB- SEQUENT EPISODE OF CARE |
| 410.70 | ACUTE MYOCARDIAL INFARCTION, SUBENDOCARDIAL INFARCTION, EPISODE OF CARE UNSPECIFIED |
| 410.71 | ACUTE MYOCARDIAL INFARCTION, SUBENDOCARDIAL INFARCTION, INITIAL EPISODE OF CARE |
| 410.72 | ACUTE MYOCARDIAL INFARCTION, SUBENDOCARDIAL INFARCTION, SUBSEQUENT EPISODE OF CARE |
| 410.8 | MYOCARDIAL INFARCT NEC* |
| 410.80 | ACUTE MYOCARDIAL INFARCTION, OF OTHER SPECIFIED SITES, EPISODE OF CARE UNSPECIFIED |
| 410.81 | ACUTE MYOCARDIAL INFARCTION, OF OTHER SPECIFIED SITES, INITIAL EPISODE OF CARE |
| 410.82 | ACUTE MYOCARDIAL INFARCTION, OF OTHER SPECIFIED SITES, SUBSEQUENT EPISODE OF CARE |
| 410.90 | ACUTE MYOCARDIAL INFARCTION, UNSPECIFIED SITE, EPISODE OF CARE UNSPECIFIED |
| 410.92 | ACUTE MYOCARDIAL INFARCTION, UNSPECIFIED SITE, SUBSEQUENT EPISODE OFCARE |
| 4109 | MYOCARDIAL INFARCTION – ACUTE |

|  | **Transient Ischemic Attack / Cerebrovascular accident** |
| --- | --- |
| 434.11 | CEREBRAL EMBOLISM WITH CEREBRAL INFARCTION |
| 434.90 | CEREBRAL ARTERY OCCLUSION, UNSPECIFIED, WITHOUT MENTION OF CEREBRAL INFARCTION |
| 434.91 | VERTEBROBASILAR ISCHEMIC SYNDROME |
| 4341 | CEREBRAL EMBOLISM |
| 4349 | CEREBELLAR INFARCTION |
| 43491 | CVA - ISCHEMIC STROKE |
| 435.9 | transient ischemic attack (TIA) |
| 4359 | TRANSIENT CEREBRAL ISCHEMIA |
| 436 | CEREBRO-VASCULAR ACCIDENT |
| 437.7 | TRANSIENT GLOBAL AMNESIA |

|  | **Systemic emboli** |
| --- | --- |
| 444.09 | OTHER ARTERIAL EMBOLISM AND THROMBOSIS OF ABDOMINAL AORTA |
| 444.1 | EMBOLISM AND THROMBOSIS OF THORACIC AORTA |
| 444.21 | ARTERIAL EMBOLISM AND THROMBOSIS OF UPPER EXTREMITY |
| 444.22 | ARTERIAL EMBOLISM AND THROMBOSIS OF LOWER EXTREMITY |
| 444.81 | EMBOLISM AND THROMBOSIS OF ILIAC ARTERY |
| 444.89 | EMBOLISM AND THROMBOSIS OF OTHER ARTERY |
| 444.9 | EMBOLISM AND THROMBOSIS OF UNSPECIFIED ARTERY |
| 44421 | EMBOLISM OF ARM |
| 44422 | EMBOLISM AND THROMBOSIS OF LEGS |
| 4448 | MESENTERC ARTERY OCCLUSION |

|  | **Bleeding** |
| --- | --- |
| 362.81 | RETINAL HEMORRHAGE |
| 376.32 | ORBITAL HEMORRHAGE |
| 377.42 | HEMORRHAGE IN OPTIC NERVE SHEATHS |
| 379.23 | VITREOUS HEMORRHAGE |
| 430 | SUBARACHNOID HEMORRHAGE |
| 431 | INTRACEREBRAL HEMORRHAGE |
| 432.0 | NONTRAUMATIC EXTRADURAL HEMORRHAGE |
| 432.9 | UNSPECIFIED INTRACRANIAL HEMORRHAGE |
| 4590 | HEMORRHAGE RETROPERITONEAL |
| 530.82 | ESOPHAGEAL HEMORRHAGE |
| 531.00 | ACUTE GASTRIC ULCER WITH HEMORRHAGE, WITHOUT MENTION OF OBSTRUCTION |
| 531.01 | ACUTE GASTRIC ULCER WITH HEMORRHAGE, WITH OBSTRUCTION |
| 531.10 | ACUTE GASTRIC ULCER WITH PERFORATION, WITHOUT MENTION OF OBSTRUCTION |
| 531.11 | ACUTE GASTRIC ULCER WITH PERFORATION, WITH OBSTRUCTION |
| 532.20 | ACUTE DUODENAL ULCER WITH HEMORRHAGE AND PERFORATION, WITHOUT MENTION OF OBSTRUCTION |
| 532.21 | ACUTE DUODENAL ULCER WITH HEMORRHAGE AND PERFORATION, WITH OBSTRUCTION |
| 532.40 | CHRONIC OR UNSPECIFIED DUODENAL ULCER WITH HEMORRHAGE, WITHOUT MENTION OF OBSTRUCTION |
| 532.41 | CHRONIC OR UNSPECIFIED DUODENAL ULCER WITH HEMORRHAGE, WITH OBSTRUCTION |
| 5320 | DUODENAL ULCER WITH HEMORRHAGE |
| 533.00 | ACUTE PEPTIC ULCER OF UNSPECIFIED SITE WITH HEMORRHAGE, WITHOUT MENTION OF OBSTRUCTION |
| 533.01 | ACUTE PEPTIC ULCER OF UNSPECIFIED SITE WITH HEMORRHAGE, WITH OBSTRUCTION |
| 534.00 | ACUTE GASTROJEJUNAL ULCER WITH HEMORRHAGE, WITHOUT MENTION OF OBSTRUCTION |
| 534.01 | ACUTE GASTROJEJUNAL ULCER, WITH HEMORRHAGE, WITH OBSTRUCTION |
| 535.01 | ACUTE GASTRITIS, WITH HEMORRHAGE |
| 535.11 | ATROPHIC GASTRITIS, WITH HEMORRHAGE |
| 535.31 | ALCOHOLIC GASTRITIS, WITH HEMORRHAGE |
| 535.41 | OTHER SPECIFIED GASTRITIS, WITH HEMORRHAGE |
| 535.51 | UNSPECIFIED GASTRITIS AND GASTRODUODENITIS, WITH HEMORRHAGE |
| 569.85 | ANGIODYSPLASIA OF INTESTINE WITH HEMORRHAGE |
| 578.9 | HEMORRHAGE OF GASTROINTESTINAL TRACT, UNSPECIFIED |
| 853.00 | OTH&UNSP INTRACRANIAL HEMORRHAGE FOLLOWING INJ WO MENTION OF OPEN INTRACRANIAL WOUND, W STATE AWA*** |
| 853.01 | OTH&UNSP INTRACRANIAL HEMORRHAGE FOLLOWING INJ. WO MENTION OF OPEN INTRACRANIAL WOUND, W NO COMA |
| 853.02 | OTH&UNSP INTRACRANIAL HEMORRHAGE FOLLOWING INJ WO MENTION OF OPEN INTRACRANIAL WOUND, W BRIEF COMA |
| 853.03 | OTH&UNSP INTRACRANIAL HEMORRHAGE FOLLOWING INJ WO MENTION OF OPEN INTRACRANIAL WOUND W MODERATE COMA |
| 853.04 | OTH&UNSP INTRACRANIAL HEMORRHAGE FOLLOWING INJ WO MENTION OF OPEN INTRACRANIAL WOUND, W PROLONGED*** |
| 853.05 | OTH&UNSP INTRACRANIAL HEMORRHAGE FOLLOWING INJ. WO MENTION OF OPEN INTRACRANIAL WOUND, W PROLONGE*** |
| 853.06 | OTH&UNSP INTRACRANIAL HEMORRHAGE FOLLOWING INJ WO MENTION OF OPEN INTRACRANIAL WOUND, W COMA OF U*** |
| 853.09 | OTH&UNSP INTRACRANIAL HEMORRHAGE FOLLOWING INJ WO MENTION OF OPEN INTRACRANIAL WOUND, W CONCUSSIO*** |
| 853.10 | OTH&UNSP INTRACRANIAL HEMORRHAGE FOLLOWING INJ. W OPEN INTRACRANIAL WOUND, W STATE OF AWARENESS UNSP |
| 853.11 | OTH&UNSP INTRACRANIAL HEMORRHAGE FOLLOWING INJ. W OPEN INTRACRANIAL WOUND, W NO LOSS OF CONSCIOUSNES |
| 853.12 | OTH&UNSP INTRACRANIAL HEMORRHAGE FOLLOWING INJ. W OPEN INTRACRANIAL WOUND, W BRIEF (UND 1 HR)COMA |
| 853.13 | OTH&UNSP INTRACRANIAL HEMORRHAGE FOLLOWING INJ W OPEN INTRACRANIAL WOUND, W MODERATE COMA, 1-24 HOUR |
| 853.14 | OTH&UNSP INTRACRANIAL HEMORRHAGE FOLLOWING INJ W OPEN INTRACRANIAL WOUND, W PROLONGED(OVER 24 HR)*** |
| 997.02 | IATROGENIC CEREBROVASCULAR INFARCTION OR HEMORRHAGE |
