# Supplementary material for: Thrombocytopenia and thrombocytosis are associated with different outcome in atrial fibrillation patients on anticoagulant therapy
Source: PLoS One. 2019 Nov 7;14(11):e0224709. doi: 10.1371/journal.pone.0224709 (PMC6837521; doi:10.1371/journal.pone.0224709)
Supplement: S2 Table — (DOCX) [file pone.0224709.s002.docx]

|  | **Rate of missing data n (%)** |
| --- | --- |
| **Total population n=11,527** |  |
|  |  |
| **Age, years** | 0 |
| **Male** | 0 |
| **Past history** |  |
| **CHF** | 0 |
| **DM** | 0 |
| **Hypertension** | 0 |
| **PVD** | 0 |
| **Past PE/DVT** | 0 |
| **IHD** | 0 |
| **Fall** | 0 |
| **Alcohol consumption** | 0 |
| **TIA/CVA** | 0 |
| **Bleeding** | 0 |
| **Aspirin** | 0 |
| **Clopidogrel** | 0 |
| **Weight, Kg** | 754 (6.5) |
| **CHADS2 score** | 0 |
| **CHA2DS2-VASC score** | 0 |
| **Laboratory results** |  |
| **EF, %** | 4283 (37) |
| **Creatinine, mg/dL** | 49 (0.4) |
| **Hb, g/dL** | 55 (0.5) |
| **WBC, 10^9^/L** | 439 (3.8) |
| **MPV, fL** | 505 (4.4) |
| **GFR, mL/min** | 49 (0.4) |

CHF= congestive heart failure; DM= diabetes mellitus; PVD= peripheral vascular disease; PE/DVT= pulmonary emboli/ deep vein thrombosis; IHD= ischemic heart disease; TIA/CVA= transient ischemic attack/ cerebrovascular accident; EF= ejection fraction; Hb= hemoglobin, WBC= white blood cells; MPV=mean platelet volume; GFR=glomerular filtration rate.
