# Supplementary material for: Thrombocytopenia and thrombocytosis are associated with different outcome in atrial fibrillation patients on anticoagulant therapy
Source: PLoS One. 2019 Nov 7;14(11):e0224709. doi: 10.1371/journal.pone.0224709 (PMC6837521; doi:10.1371/journal.pone.0224709)
Supplement: S3 Table — (DOCX) [file pone.0224709.s003.docx]

|  | **Low platelet** | |  | **Normal platelets** | |  | **High platelet** | |
| --- | --- | --- | --- | --- | --- | --- | --- | --- |
| **Outcome** | **NOAC**  **n=712** | **Warfarin**  **n=905** |  | **NOAC**  **n=4467** | **Warfarin**  **n=5272** |  | **NOAC**  **n=61** | **Warfarin**  **n=110** |
| **Mortality n (%)** | 156 (22) | 418 (46) |  | 740 (17) | 2075 (39) |  | 19 (31) | 49 (45) |
| **MI n (%)** | 18 (2.5) | 25 (2.7) |  | 88 (1.9) | 166 (3.1) |  | 3 (4.9) | 7 (6.3) |
| **TIA/CVA n (%)** | 20 (2.8) | 30 (3.3) |  | 144 (3.2) | 306 (5.8) |  | 1 (1.6) | 7 (6.3) |
| **Systemic Emboli n (%)** | 6 (0.8) | 13 (1.4) |  | 26 (0.6) | 77 (1.4) |  | 2 (3.2) | 1 (0.9) |
| **Bleeding n (%)** | 25 (3.5) | 65 (7.2) |  | 127 (2.8) | 266 (5) |  | 3 (4.9) | 5 (4.5) |
| **Combined-1 n (%)** | 195 (27) | 458 (51) |  | 959 (21) | 2321 (44) |  | 22 (36) | 53 (48) |
| **Combined-2 n (%)** | 65 (9.1) | 110 (12) |  | 341 (7.6) | 671 (13) |  | 7 (11) | 18 (16) |

NOAC= non-vitamin K antagonist oral anticoagulants; MI= myocardial infarction; TIA/CVA= transient ischemic attack/ cerebrovascular accident; Combined-1 includes: mortality, MI, TIA/CVA, systemic emboli and bleeding; Combined-2 includes: MI, TIA/CVA, systemic emboli and bleeding.
