# Supplementary material for: Thrombocytopenia and thrombocytosis are associated with different outcome in atrial fibrillation patients on anticoagulant therapy
Source: PLoS One. 2019 Nov 7;14(11):e0224709. doi: 10.1371/journal.pone.0224709 (PMC6837521; doi:10.1371/journal.pone.0224709)
Supplement: S4 Table — (DOCX) [file pone.0224709.s004.docx]

|  | **Low platelet** | **Normal platelet** |  |
| --- | --- | --- | --- |
|  | **NOAC** | **NOAC** | **p value** |
|  | **n=712** | **n=4467** |  |
| **Age, years** | 79.7 (71.9, 85.5) | 77.7 (69.5, 84.4) | **<0.001** |
| **Male** | 509 (71.5) | 2221 (49.7) | **<0.001** |
| **Medical history** |  |  |  |
| **CHF** | 259 (36.4) | 1375 (30.8) | **0.003** |
| **DM** | 242 (34) | 1486 (33.3) | 0.73 |
| **Hypertension** | 509 (71.5) | 3112 (69.7) | 0.33 |
| **PVD** | 49 (6.9) | 258 (5.8) | 0.28 |
| **Past DVT/PE** | 27 (3.8) | 202 (4.5) | 0.43 |
| **IHD** | 363 (51) | 1872 (41.9) | **<0.001** |
| **Fall** | 93 (13.1) | 634 (14.2) | 0.45 |
| **Alcohol** | 24 (3.4) | 106 (2.4) | 0.12 |
| **TIA/CVA** | 183 (25.7) | 1259 (28.2) | 0.18 |
| **Bleeding** | 72 (10.1) | 400 (9) | 0.33 |
| **Aspirin** | 245 (34.4) | 1543 (34.5) | 0.97 |
| **Clopidogrel** | 92 (12.9) | 588 (13.2) | 0.91 |
| **Weight Kg** | 76 (68, 89) | 76 (66, 88) | 0.34 |
| **CHADS_2_ score** | 3 (2, 4) | 2 (1, 4) | 0.055 |
| **CHA_2_DS_2_-VASC score** | 4 (3, 5) | 4 (3, 6) | 0.93 |
| **Laboratory** |  |  |  |
| **EF %** | 55 (42, 60) | 60 (50, 60) | **<0.001** |
| **Creatinine, mg/dL** | 1 (0.9, 1.3) | 1 (0.8, 1.2) | **<0.001** |
| **Hb g/dL** | 12.4±1.8 | 12.4±1.8 | 0.45 |
| **WBC 10^9^/L** | 7.2 (5.8, 9) | 8.8 (7.1, 11) | **<0.001** |
| **MPV fL** | 9.7 (8.9, 10.7) | 8.8 (8.1, 9.7) | **<0.001** |
| **GFR mL/min** | 64 (49.3, 81.6) | 67.8 (52.5, 84.9) | **0.01** |

CHF= congestive heart failure; DM= diabetes mellitus; PVD= peripheral vascular disease; PE/DVT= pulmonary emboli/ deep vein thrombosis; IHD= ischemic heart disease; TIA/CVA= transient ischemic attack/ cerebrovascular accident; EF= ejection fraction; Hb= hemoglobin, WBC= white blood cells; MPV=mean platelet volume; GFR=glomerular filtration rate.
