# Supplementary material for: Thrombocytopenia and thrombocytosis are associated with different outcome in atrial fibrillation patients on anticoagulant therapy
Source: PLoS One. 2019 Nov 7;14(11):e0224709. doi: 10.1371/journal.pone.0224709 (PMC6837521; doi:10.1371/journal.pone.0224709)
Supplement: S5 Table — (DOCX) [file pone.0224709.s005.docx]

| **Outcome** |  |  |  |
| --- | --- | --- | --- |
|  | **HR** | **95 CI** | **p value** |
| **Mortality** | 1.15 | 0.96-1.38 | 0.12 |
| **MI** | 1.11 | 0.66-1.87 | 0.68 |
| **TIA/CVA** | 0.85 | 0.52-1.39 | 0.5 |
| **Systemic emboli** | 1.13 | 0.43-3 | 0.8 |
| **Bleeding** | 1.1 | 0.7-1.74 | 0.66 |
| **Combined-1** | 1.12 | 1-1.38 | **0.047** |
| **Combined-2** | 1.07 | 0.8-1.4 | 0.67 |

MI= myocardial infarction; TIA/CVA= transient ischemic attack/ cerebrovascular accident; Combined-1 includes: mortality, MI, TIA/CVA, systemic emboli and bleeding; Combined-2 includes: MI, TIA/CVA, systemic emboli and bleeding.
