# Supplementary material for: Thrombocytopenia and thrombocytosis are associated with different outcome in atrial fibrillation patients on anticoagulant therapy
Source: PLoS One. 2019 Nov 7;14(11):e0224709. doi: 10.1371/journal.pone.0224709 (PMC6837521; doi:10.1371/journal.pone.0224709)
Supplement: S6 Table — (DOCX) [file pone.0224709.s006.docx]

|  | **High platelet** | **Normal platelet** |  |
| --- | --- | --- | --- |
|  | **NOAC** | **NOAC** | **p value** |
|  | **n=61** | **n=4467** |  |
| **Age in years** | 79.5 (70.6, 85.5) | 77.7 (69.5, 84.4) | 0.15 |
| **Male** | 27 (44.3) | 2221 (49.7) | 0.44 |
| **Past history** |  |  |  |
| **CHF** | 21 (34.4) | 1375 (30.8) | 0.58 |
| **DM** | 29 (47.5) | 1486 (33.3) | **0.02** |
| **Hypertension** | 53 (86.9) | 3112 (69.7) | **0.005** |
| **PVD** | 5 (8.2) | 258 (5.8) | 0.58 |
| **Past DVT/PE** | 5 (8.2) | 202 (4.5) | 0.2 |
| **IHD** | 27 (44.3) | 1872 (41.9) | 0.79 |
| **Fall** | 8 (13.1) | 634 (14.2) | 0.86 |
| **Alcohol** | 0 (0) | 106 (2.4) | 0.4 |
| **TIA/CVA** | 17 (27.9) | 1259 (28.2) | 1 |
| **Bleeding** | 4 (6.6) | 400 (9) | 0.65 |
| **Aspirin** | 17 (27.9) | 1543 (34.5) | 0.28 |
| **Clopidogrel** | 10 (16.4) | 588 (13.2) | 0.57 |
| **Weight** | 71 (60.5, 87.7) | 76 (66, 88) | **0.02** |
| **CHADS_2_ score** | 3 (2, 4) | 2 (1, 4) | **0.02** |
| **CHA_2_DS_2_-VASC score** | 5 (3, 6) | 4 (3, 6) | **0.04** |
| **Laboratory** |  |  |  |
| **EF %** | 50 (45, 60) | 60 (50, 60) | 0.054 |
| **Creatinine mg/dL** | 0.8 (0.7, 1.2) | 1 (0.8, 1.2) | **0.01** |
| **Hb g/dL** | 10.9±1.5 | 12.4±1.8 | **0.004** |
| **WBC 109/L** | 11.1 (8.3, 14.4) | 8.8 (7.1, 11) | **<0.001** |
| **MPV fL** | 7.8 (7.1, 8.5) | 8.8 (8.1, 9.7) | **<0.001** |
| **GFR mL/min** | 74.3 (53.8, 104.5) | 67.8 (52.5, 84.9) | 0.051 |

CHF= congestive heart failure; DM= diabetes mellitus; PVD= peripheral vascular disease; PE/DVT= pulmonary emboli/ deep vein thrombosis; IHD= ischemic heart disease; TIA/CVA= transient ischemic attack/ cerebrovascular accident; EF= ejection fraction; Hb= hemoglobin, WBC= white blood cells; MPV=mean platelet volume; GFR=glomerular filtration rate.
