# Supplementary material for: Thrombocytopenia and thrombocytosis are associated with different outcome in atrial fibrillation patients on anticoagulant therapy
Source: PLoS One. 2019 Nov 7;14(11):e0224709. doi: 10.1371/journal.pone.0224709 (PMC6837521; doi:10.1371/journal.pone.0224709)
Supplement: S7 Table — (DOCX) [file pone.0224709.s007.docx]

| **Outcome** |  |  |  |
| --- | --- | --- | --- |
|  | **HR** | **95 CI** | **p value** |
| **Mortality** | 1.54 | 0.97-2.45 | 0.07 |
| **MI** | 2.6 | 0.82-8.27 | 0.1 |
| **CVA** | 0.42 | 0.06-3.03 | 0.39 |
| **Systemic emboli** | 7.07 | 1.66-30.25 | **0.008** |
| **Bleeding** | 1.41 | 0.44-4.46 | 0.56 |
| **Combined-1** | 1.48 | 0.96-2.26 | 0.07 |
| **Combined-2** | 1.54 | 0.72-3.3 | 0.27 |

MI= myocardial infarction; TIA/CVA= transient ischemic attack/ cerebrovascular accident; Combined-1 includes: mortality, MI, TIA/CVA, systemic emboli and bleeding; Combined-2 includes: MI, TIA/CVA, systemic emboli and bleeding.
